# Simple and cost-effective method of highly conductive and elastic carbon nanotube/polydimethylsiloxane composite for wearable electronics

**DOI:** 10.1038/s41598-017-18209-w

**Published:** 2018-01-22

**Authors:** Jeong Hun Kim, Ji-Young Hwang, Ha Ryeon Hwang, Han Seop Kim, Joong Hoon Lee, Jae-Won Seo, Ueon Sang Shin, Sang-Hoon Lee

**Affiliations:** 1 KU-KIST Graduate School of Converging Science and Technology, Korea University, Seoul, 02841 Republic of Korea; 2 Department of Biomedical Engineering, College of Health Science, Korea University, Seoul, 02841 Republic of Korea; 3 Department of Nanobiomedical Science and BK21 Plus NBM Global Research Center for Regenerative Medicine, Dankook University, Cheonan, 31116 Republic of Korea; 4 Present Address: International Carbon Research Institute, Korea Institute of Carbon Convergence Technology 110‐11 Banryong‐ro Deokjin‐gu, Jeonju, 54853 Republic of Korea

## Abstract

The development of various flexible and stretchable materials has attracted interest for promising applications in biomedical engineering and electronics industries. This interest in wearable electronics, stretchable circuits, and flexible displays has created a demand for stable, easily manufactured, and cheap materials. However, the construction of flexible and elastic electronics, on which commercial electronic components can be mounted through simple and cost-effective processing, remains challenging. We have developed a nanocomposite of carbon nanotubes (CNTs) and polydimethylsiloxane (PDMS) elastomer. To achieve uniform distributions of CNTs within the polymer, an optimized dispersion process was developed using isopropyl alcohol (IPA) and methyl-terminated PDMS in combination with ultrasonication. After vaporizing the IPA, various shapes and sizes can be easily created with the nanocomposite, depending on the mold. The material provides high flexibility, elasticity, and electrical conductivity without requiring a sandwich structure. It is also biocompatible and mechanically stable, as demonstrated by cytotoxicity assays and cyclic strain tests (over 10,000 times). We demonstrate the potential for the healthcare field through strain sensor, flexible electric circuits, and biopotential measurements such as EEG, ECG, and EMG. This simple and cost-effective fabrication method for CNT/PDMS composites provides a promising process and material for various applications of wearable electronics.

## Introduction

Recent progress in wireless communication, Internet of Things (IoT) devices, and biomedical engineering have enabled continuous monitoring of mental and physical health, which is one of the most important issues for ubiquitous healthcare using mobile devices. For such purposes, highly stretchable, flexible, and biocompatible electronics are of great interest. In recent decades, some electronic skin materials have been developed, demonstrating potential applications in wearable electronics, flexible and stretchable circuits, flexible displays and energy storage devices, and electronic skins^1–10^. Most electronic skins were developed through new processes involving innovative design concepts with established materials or by fabricating devices with newly developed stretchable composites. However, the construction of skin-like electronics on which commercial electronic components can be mounted by a simple and cost-effective process remains challenging. The development of such construction processes would shift flexible electronics to a new technical paradigm.

Various nanocomposite materials blending nanomaterials and elastic polymers have recently attracted attention for such flexible electronics. It is well known that carbon based nano-filler, namely, CNTs can improve mechanical properties and electrical and thermal conductivity of polymer composites, due to their extraordinary advantages such as a high aspect ratio, superior elastic modulus, and high conductivity^11–15^. In addition, low manufacturing cost with mass production make CNTs an ideal candidate filler. Hence, we also considered and used CNTs as a filler in this study. However, producing CNT nanocomposite materials in viscoelastic polymer solutions is challenging because the CNTs tend to become severely agglomerated. If CNT particles remain heterogeneous, the electrical conductivity can be severely compromised, impeding the use of CNTs for skin-like electronics. Several methods to promote the dispersion of nanoparticles have been developed, including surface modification, shear mixing, mechanical agitation, ultrasonic treatment, and ball- or micro-bead milling^16–19^. For examples, Sekitani *et al*.^10^ reported a fluorinated copolymer/SWNT composite with remarkable properties (a high conductivity of 57 S/cm, elongation up to 134%) using the ionic liquid, and Velasco-Santos *et al*.^20^ incorporated 1-wt% chemically functionalized MWNTs into a polymer matrix by *in situ* polymerization and reported that the storage modulus increased by 1135% compared to existing similar composites. Fukushima *et al*.^21^ produced CNT/polymer nanocomposites by the free-radical polymerization of an imidazolium ion-based ionic liquid bearing a methacrylate group to prevent decrease of mechanical modulus as the content of CNTs increased. It is difficult to develop a simple and cost-effective method for homogeneous dispersion of CNTs in a matrix, as well as prevents the reaggregation of dispersed CNTs for extended periods. Furthermore, the mass production of CNT nanocomposite materials with good dispersion in various polymer matrices could become important for extensive applications of stretchable electronics.

In this study, we propose a simple, fast, and cost-effective fabrication method for a homogenously hybridized CNT/polydimethylsiloxane (PDMS) composite with high conductivity, stretchability, and flexibility. To our knowledge, the flexible and stretchable digital circuit containing only CNT/PDMS conductive lines and commercial electronic components demonstrated here is the first of its kind.

## Results

### Principles and Process of Homogenous Dispersion of CNTs in PDMS

CNTs are highly entangled by van der Waals forces, and the presence of CNTs in large bundles or dense agglomerates can cause uncontrolled electronic alterations and poor performance^22–24^. Therefore, creating homogeneous distributions of CNTs in the PDMS polymer matrix is necessary for obtaining high electrical performance from CNT/PDMS devices. Non-covalent functionalization preserves the intrinsic electrical properties of the outer CNT surface, unlike covalent CNT surface modification^25^. Constructing non-covalent functionalized CNTs requires the employment of a good dispersion solvent that possesses both amphiphilic characteristics and relatively small-size molecules. The hydrophobic regions of the solvent molecules could individually detach CNT fibers through non-polar interactions with the carbonaceous CNT walls, while the hydrophilic regions could stabilize the hybridized CNT/PDMS solution^26,27^. Even though a solvent could fulfill all of these requirements and form a good initial dispersion of CNTs, the CNT fibers may reaggregate over time. Therefore, the selection of an appropriate solvent is important in determining the quality and the mechanical and electrical characteristics of the final CNT/PDMS nanohybrid.

Nonpolar solvents (e.g., toluene and hexane) were not usable because they could swell the PDMS matrix. Aprotic polar solvents (e.g., dichloromethane and chloroform) were not usable because they were highly volatile, even at room temperature, and would entrap air pockets within the CNT/PDMS nanohybrid, which could negatively affect the mechanical and electrical properties of the composite. Alcohol solvents have aliphatic chains in the hydrophobic region and hydroxyl groups in the hydrophilic region, allowing the dispersion of CNTs with a stable composite matrix. However, alcohols with smaller chain lengths, such as methyl alcohol and ethyl alcohol, exhibit a low affinity with CNTs because of their relatively short hydrophobic regions^28–30^.

**Figure 1:**
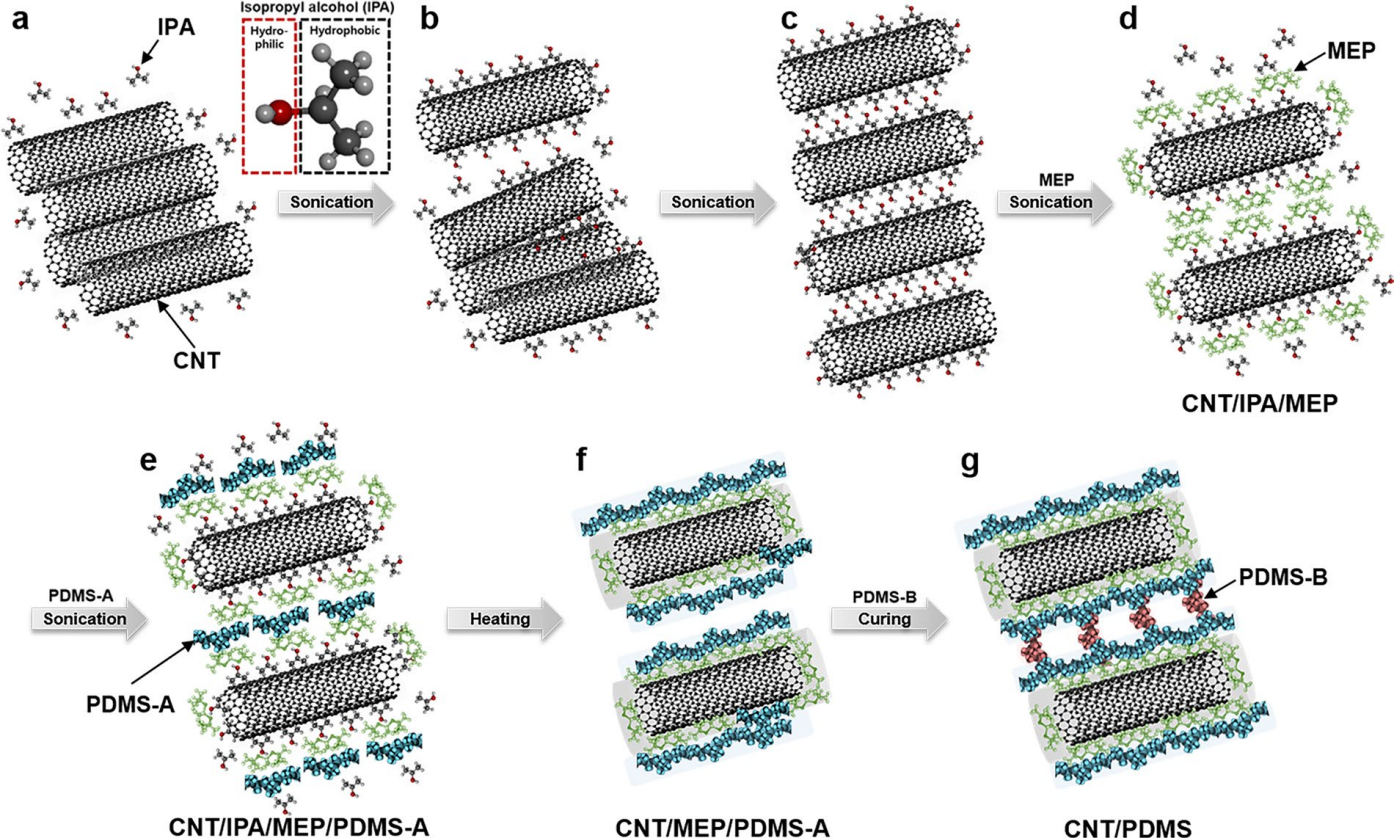
Schematics of CNT/PDMS hybrid nanocomposite fabrication. (**a**–**c**) Detachment and dispersion of aggregated CNT bundles in IPA by hydrophobic regions of IPA and ultrasonication, (**d**) wrapping of IPA-attached CNTs by MEP, (**e**) attachment of PDMS-A to MEP, (**f**) evaporation of IPA by heating, followed by entanglement of CNT/PDMS complexes, and (**g**) fabrication of CNT/PDMS composite materials after mixing with curing agent for cross-linking of PDMS.

We selected isopropyl alcohol (IPA) as a suitable solvent because both CNTs and PDMS are partially soluble in IPA^28^. IPA is a colorless and volatile organic liquid. Because IPA has a relatively large surface tension and twice the vapor density of air, air bubbles are easily removed from the solvent. IPA has an amphiphilic structure comprising three hydrocarbon units and one hydroxyl group. The hydrophobic part is easily attached to the highly hydrophobic CNT surfaces, forming IPA-coated CNTs with hydroxyl groups located on the outer layer of the carbonaceous nanotube complexes (CNT/IPA complexes). The complexes can interact with each other and with IPA solvent molecules via the hydrophilic hydroxyl groups (Fig. 1a).

When CNTs and IPA are mixed and sonicated, strongly aggregated CNT bundles are temporarily separated by the physical force exerted by the ultrasound source. The separated CNT portions and the gaps between them are then coated and filled with IPA. The gaps are widened with additional sonication causing complete separation of the individual CNT/IPA complexes and their stabilization in the IPA solvent (Fig. 1b and c). Generally, strong sonication is used for CNT dispersion to overcome the van der Waals forces between adhered tubes within CNT assemblies. However, strong sonication can mechanically damage the CNTs by exposing them to excessive stress and heat, thereby shortening the length and significantly decreasing the electrical conductivity of the tubes^31–33^. In this study, sonication was performed at the high frequency of 40 kHz and the relatively low power of ~80–100 W for less than 30 min in order to preserve the CNT integrity.

Before blending the CNT-dispersed IPA solution with PDMS, a small amount of methyl group-terminated PDMS (MEP) with low viscosity (100 cSt) was added to the CNT-dispersed solution and blended for 10 min by sonication. MEP is a non-volatile polymeric organosilicon material consisting of –(CH3)2SiO– structural units. MEP penetrates the IPA phase in individual CNT/IPA complexes and adheres to the hydrophobic CNT surface. Then, thermodynamically stable complexes of CNT/IPA/MEP, coated with IPA/MEP, are formed in the IPA solution (Fig. 1d). The base of the Sylgard 184 silicone elastomer kit (PDMS-A, viscosity of 3500 cSt) was blended with the CNT/IPA/MEP solution by sonication. Here, PDMS-A could make direct contact with the MEP phase surrounding the CNT tubes, and both PDMS-A and CNT/MEP became stable and homogenized as CNT/IPA/MEP/PDMS-A units in the IPA solution (Fig. 1e).

In next step, IPA (boiling point of 82.6 °C) was vaporized slowly and completely using a 50–60 °C hot plate, leaving neither bubbles nor air pockets among the CNT/MEP/PDMS nanohybrid units. If the evaporation were performed at a temperature exceeding 70 °C, rough surfaces with holes would be observed. After the complete removal of the IPA components from the CNT/IPA/MEP/PDMS-A units in IPA solution, only the CNT/MEP/PDMS units remained, forming nano-hybridized CNT/MEP/PDMS components in liquid. This MEP layer-mediated formation of the homogeneous CNT/PDMS nanohybrid material is defined as the MEP functionalization of CNT surfaces by non-covalent binding (Fig. 1f); the product is used to create the CNT/PDMS nanohybrid. Lastly, the curing agent from the Sylgard 184 silicone elastomer kit, named PDMS-B, comprising a platinum-containing compound as a curing catalyst, is added to the nanohybrid and mixed. After curing at 80 °C on a hot plate for 2 h, excellent CNT/PDMS nanocomposite materials similar to silicone rubber with well-dispersed CNTs are obtained (Fig. 1g).

In addition, we confirmed the existence of IPA, an organic solvent, at the final step. To synthesize 8-wt% CNT/PDMS, we prepared 0.8 g of CNTs, 2.01 g of MEP, 8.01 g of PDMS, and a sufficient amount of IPA. We conducted ultrasonication mixing at each step. After complete evaporation of IPA, we could confirm the weight of 10.81 g as shown in Figure S1 (the sum of the included components, except IPA, 0.8 g CNTs + 2.01 g MEP + 8.01 g PDMS = 10.82 g). This result means that IPA, an organic solvent, was removed from the final composite.

### Characterization of the CNT/PDMS composite

#### Dispersion Stability

**Figure 2:**
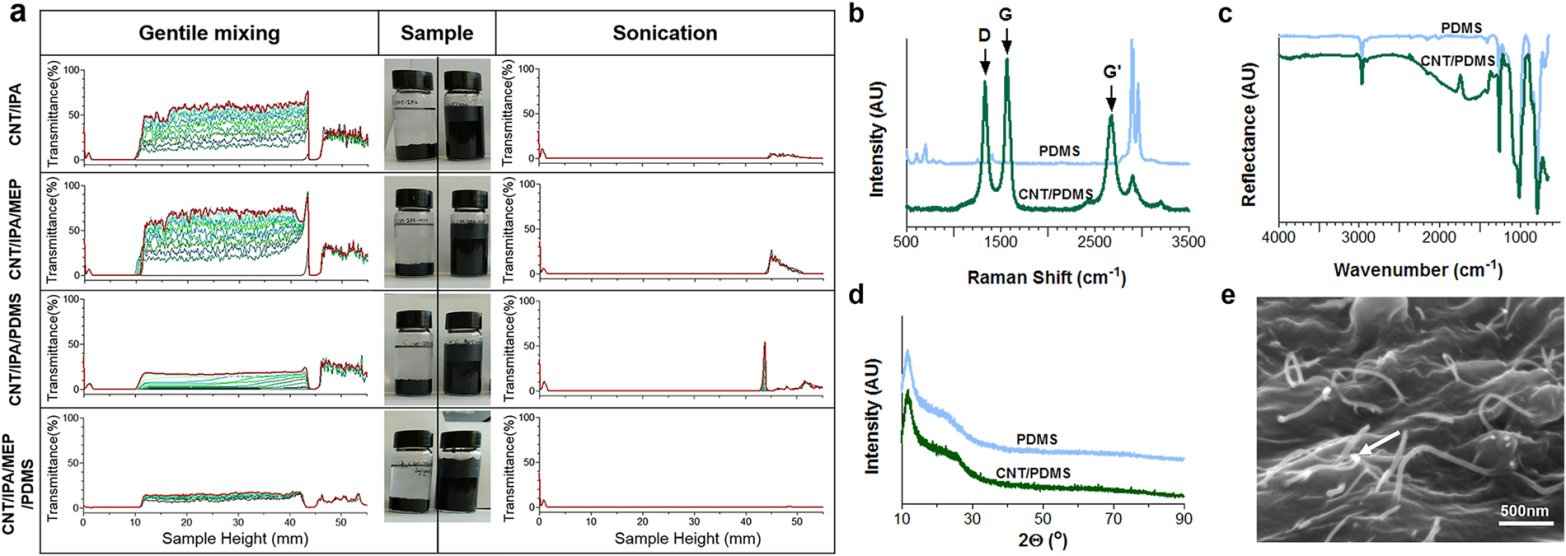
Characteristics of 4-wt% CNT/PDMS solutions and films. (**a**) Stability analysis of CNT dispersion using a TurbiScan analyzer; dispersion of CNTs in IPA (i) itself, (ii) with MEP, (iii) with MEP and PDMS-A, and (iv) with MEP and PDMS-A and -B. Data of the solutions after gentle (non-sonicated) mixing are shown in the left panel, while data after sonication are in the right panel. Photographs show the dispersion conditions of the CNT solutions two months after mixing. (**b**) Raman spectra. Light blue solid lines represent neat PDMS films; green solid lines represent CNT/PDMS films. (**c**) ATR-FTIR spectrum, (**d**) XRD data, and (**e**) representative cross-sectional SEM image of CNT/PDMS films. Arrows indicate connected CNTs.

We characterized the dispersion stability of the various CNT nanohybrid solutions using a TurbiScan stability analyzer at each mixing step before and after sonication treatment. Figure 2a shows the delta transmittance profiles obtained from various dispersions before and after sonication over time. The transmittance values of the CNT dispersions approach zero throughout the sample bottle after sonication, indicating homogeneous and stable dispersions of CNTs, CNT/MEP, CNT/MEP/PDMS-A, and CNT/MEP/PDMS-B in IPA (right panels in Fig. 2a). On the other hand, the transmittance values of the CNT dispersions without sonication treatment are significantly higher in the upper portion of the sample bottle, suggesting rapid precipitation of CNT agglomerates (left panels in Fig. 2a). Thus, the sonication process and MEP are important in forming complete dispersions.

In previous researches^34–36^, the glass transition temperature (Tg) was reported that related to the dispersion and interaction of the composite. Hence, the differential scanning calorimetry (DSC) was conducted to observe dispersion of the CNT/PDMS in this study. Before sonication in the IPA solution, the values of the Tg for the CNT, CNT/MEP, CNT/PDMS-A, and CNT/PDMS-A and -B occurred at 75.89 ± 3.55 °C, 82.70 ± 0.66 °C, 81.91 ± 0.69 °C, and 82.29 ± 1.10 °C, respectively, while the values of Tg are shifted to 80.32 ± 1.06 °C, 85.45 ± 2.32 °C, 87.81 ± 4.96 °C, and 86.05 ± 1.03 °C, respectively, after sonication. Therefore, the CNT surface configuration is important in determining the glass transition behavior of PDMS matrices: the temperature of the α-transition of the well-dispersed CNT/PDMS composites is higher than that for poor dispersions.

The Raman spectra of the PDMS and CNT/PDMS films demonstrate the symmetric structure of CNTs, quantitatively and qualitatively characterizing the carbon nanocomponents (Fig. 2b). The D-peak and G-peak are characteristic of sp2-hybridized carbon materials. The D-band peak at 1350 cm^−1^ is attributed to disordered graphite structures, or sp3-hybridized carbon atoms within the CNTs, while the high-frequency G-band peak at 1580 cm^−1^ corresponds to the structural intensity of the sp2-hybridized carbon atoms. The peak intensity increases proportionally with CNT concentration. Other peaks in the Raman spectra are attributed to the PDMS structure, which has neither D- nor G- peaks. The wide peak observed in the G band indicates disentanglement of CNTs and their subsequent dispersion in the PDMS matrix. The intensity ratio of the D and G bands is a strong indicator of the structural arrangement, known from their chirality. The sharp shapes of the G and G′peaks possibly indicate metallic-type conductivity.

The attenuated total reflection–Fourier transform infrared spectroscopy (ATR-FTIR) spectra of CNT/PDMS films demonstrate typically strong C–C and C=C broad stretching peaks at 500 to 2000 cm^−1^, respectively, indicating a high percentage of CNTs on the surfaces (Fig. 2c). The intensity of the peak observed at 1000 cm^−1^ decreased with increasing concentration of CNTs. In contrast, the peak at 1000 cm^−1^ increased with PDMS, corresponding to Si–C bond formation. Moreover, with increasing CNT percentage in PDMS, the rocking peaks of –Si(CH3)2 decrease, coinciding with an increase in the Si–H peak intensity. A significant difference between the pure PDMS and CNT/PDMS composite spectra was observed at 1000 cm^−1^, for which the peak became sharper and gained a notably lower intensity as the concentration of CNT increased in PDMS.

The X-ray diffraction (XRD) data for PDMS and CNT/PDMS composites are shown in Fig. 2d, exhibiting relatively good crystalline structures. The CNT/PDMS composites show slight increases in diffraction peak intensities at approximately 20°, indicating a greater presence of carbon core structures in the composites. The PDMS film exhibits two broad peaks at the 2° angles of ~21° and ~26°. When 4-wt% CNT is incorporated into the PDMS, the peak at 20° becomes more intense than that of PDMS, possibly from the superimposition of peaks from PDMS and the CNTs. The decreased PDMS crystallinity causes the XRD crystal peak to broaden.

The representative scanning electron microscopy (SEM) images in Fig. 2e show cross-sectional views of the CNT/PDMS film. Compared to previous reports, the micrographs indicate excellent dispersion quality, especially for a nanocomposite containing more than 4-wt% CNTs^37^. As expected, the CNT nanofiller complexes are individually buried, which is not possible for IPA- and MEP-wrapped CNTs. In addition, the CNTs form a percolation network, suggesting enhancement of electrical and mechanical properties. No free CNTs were observed on the CNT/PDMS composite surface, indicating that they fully infiltrated the CNT/PDMS film. Because direct contact between human cells and CNTs is improbable, a biocompatible CNT/PDMS film could be fabricated. In fact, the CNT/PDMS film was adhered to human skin and detached after 1 h; no CNT debris was observed (Figure S2c and d).

**Figure 3:**
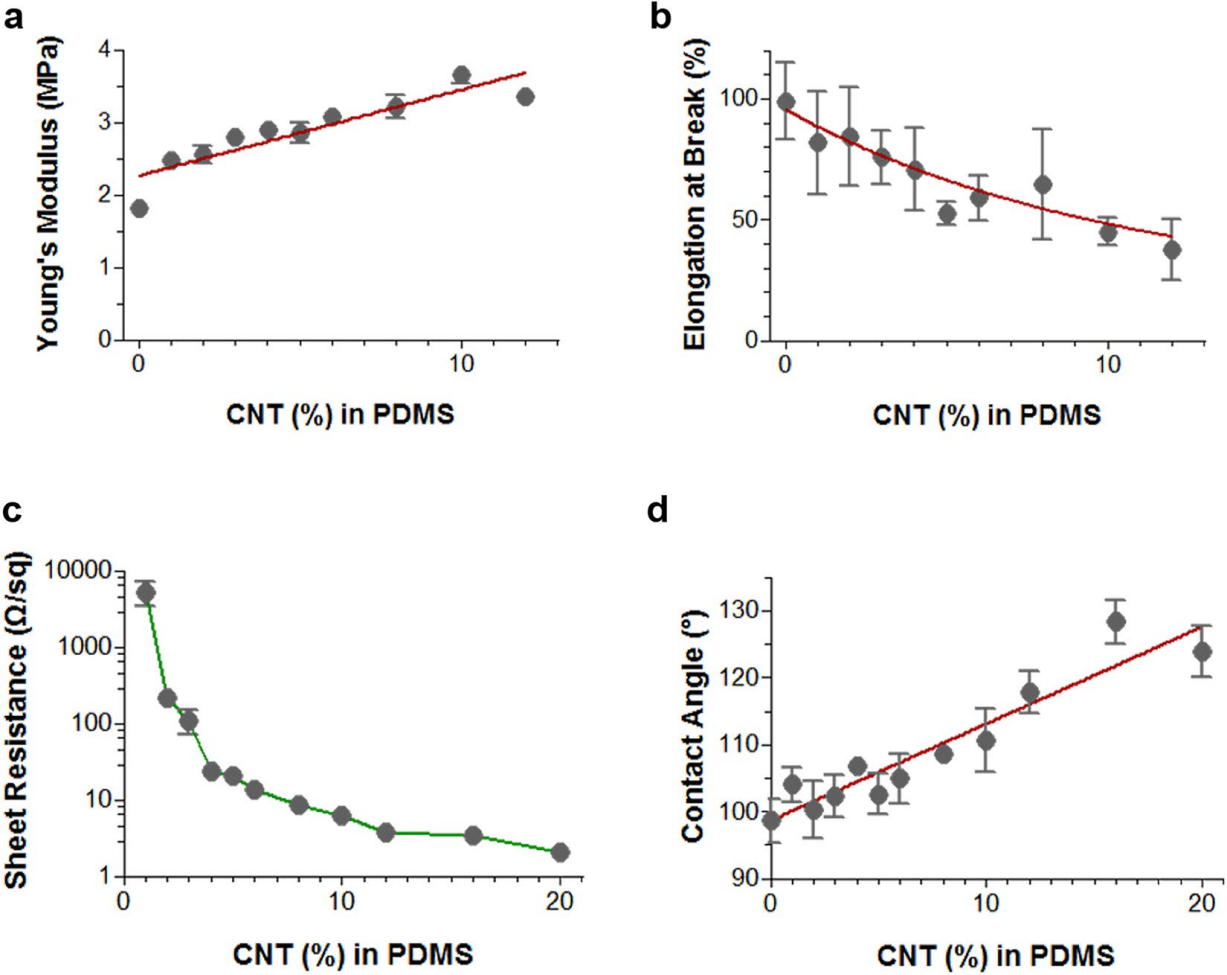
Characteristics of CNT/PDMS films for various percentiles of CNTs in PDMS (1–20 wt%). Tensile testing results for (**a**) Young’s modulus and (**b**) elongation at break. (**c**) Sheet resistance measurements for electrical conductivity, and (**d**) contact angle measurements for surface wettability. Error bars represent standard deviations (n = ~3–6). Red bold lines indicate curves fitting the data.

To observe the change of surface tension by the CNTs, the contact angle measurement was performed by establishing the angle of a liquid drop with a solid surface at the base^38^. In Fig. 3d, the contact angle of the composite increases as the content of CNTs increases because the CNTs was more exposed on the surface of the composite. We also prepared samples at each synthesis step, and measured the contact angles to compare the influence of the components. The contact angle of the CNT/PDMS film was 108.2 ± 4.1°, and those of the neat PDMS and MEP/PDMS films were 102.2 ± 1.3° and 98.6 ± 3.3°, respectively. (Table S2) These results corresponded to more hydrophobic surfaces induced by the CNTs.

In our study, we employed solution mixing which is widely used method for processing CNT/polymer nanocomposites^15^. However, unlike other methods, we added only methyl-terminated PDMS (MEP) as a mediator between the CNTs and PDMS, resulting in homogenously hybridized CNT/PDMS composite with high conductivity, stretchability, flexibility, and biocompatibility. In other words, our approach is more simple, fast, and cost-effective fabrication than previous reports.

#### Mechanical and Electrical Properties

The mechanical properties of CNT/PDMS films with different CNT concentrations were investigated to assess the elasticity of the CNT/PDMS films. The effect of the CNT/PDMS composition on the Young’s modulus and yield strain is shown in Fig. 3a and b, respectively. Notably, CNT/PDMS shows decreases in tensile strength from 7.89 ± 2.33 MPa to 4.33 ± 0.28 MPa and in yield strain from 81.84 ± 20.55% to 37.62 ± 12.39% with increases in the CNT concentrations from 1 to 12 wt%. The CNT nanofiller may interrupt the regular three-dimensional packing of PDMS molecules. The mechanical properties decrease with increased concentrations of CNTs, but remain acceptable for use in many applications. However, some researches have suggested a solution for the mechanical modulus. Mittal *et al*.^15^ reported that a homogenous dispersion and alignment prevents agglomeration and gives better load transfer to the filler material, which results in better mechanical properties. In this dispersion approach, owing to the strong interfacial interaction, the slipping of entrapped polymer molecules was suppressed, and intertubular carrier transport was also facilitated.

**Figure 4:**
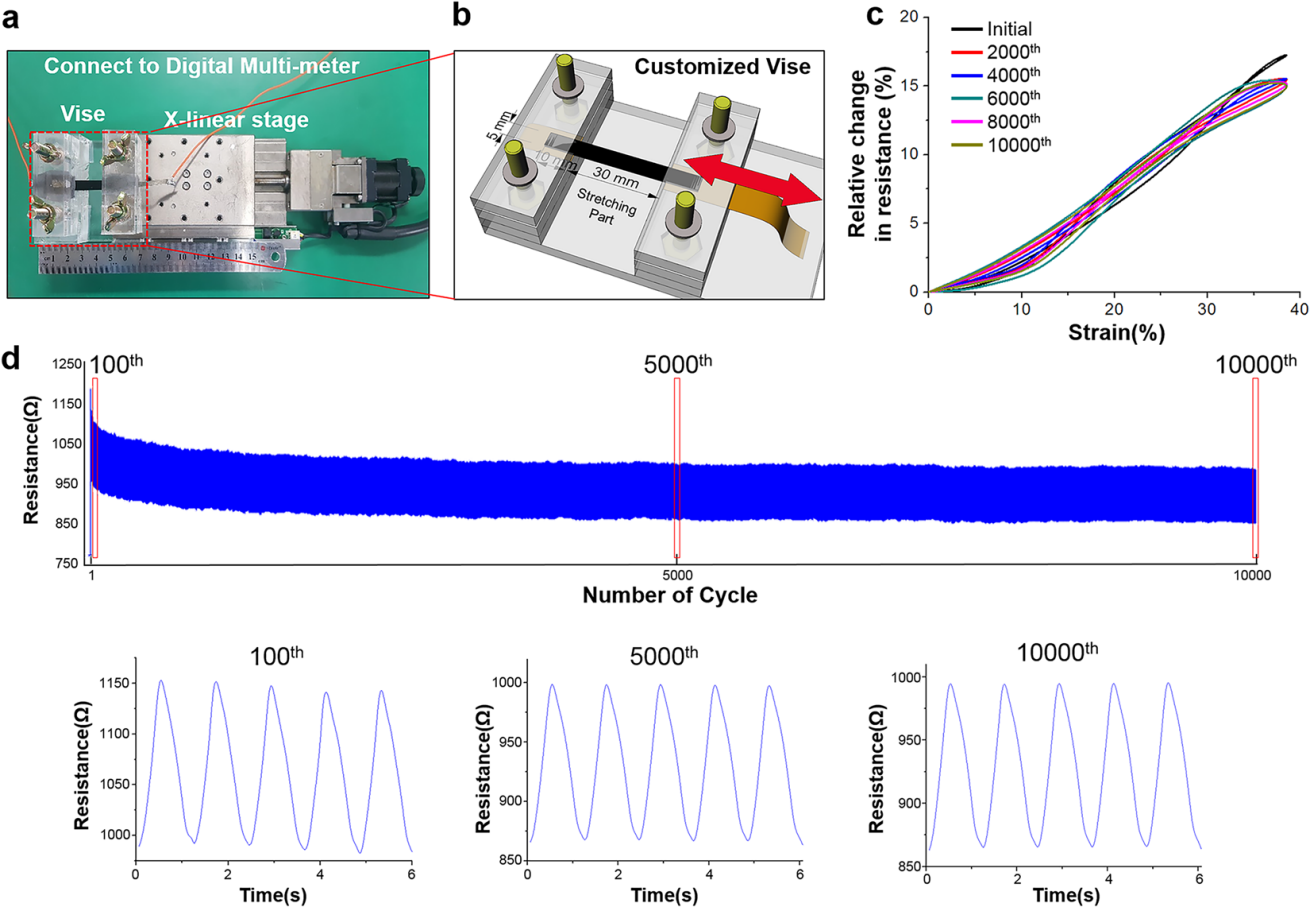
Strain cycling test. (**a**) Experimental setup and (**b**) customized vise. (**c**) Relative resistance change every 2000th cycle from the 10,000-cycle test with 4-wt% CNT/PDMS. (**d**) Change in resistance during 10,000-cycle test with 4-wt% CNT/PDMS. Graphs below represent five cycles around the 100th, 5000th, and 10000th cycle.

Figure 4a shows the electrical properties of CNT/PDMS films prepared with different CNT concentrations. The electrical resistances for the films decrease exponentially from 5225 ± 1755 to 2.03 ± 0.16 Ω/sq. Films with higher CNT contents show better electrical conductivity, but the electrical resistance saturates for samples containing more than 4-wt% CNTs. Well-dispersed CNTs in the PDMS elastomer matrix may provide efficient electrical properties for the CNT/PDMS composite, preserving connections in the conductive percolated CNT networks even after stretching, which is necessary for electrical applications.

To demonstrate the electric stability, mechanical tensile cycling tests over 10,000 times were performed on CNT/PDMS specimens with 4, 8, and 12 wt% CNTs. As shown in Fig. 4, the overall graphs of each test have similar shapes. In addition, the data for 8 and 12 wt% exhibit similar results with 4 wt% except for the initial cycle, as shown in Figure S6. The hysteresis by the initial strain was affected by the interaction (Figure S5) between CNTs in the composite. Stable electric properties were observed for all CNT/PDMS film compositions. Most CNT-based sensors show performance hysteresis under mechanical stresses^39–42^. The first test cycle of each specimen shows hysteresis because of the nanostructured network, the piezoresistive effects of CNT^43^, and the hysteresis of the PDMS^44^. As the number of cycles increase, the CNTs become stabilized in the stretched PDMS. Moreover, we confirmed the existence of residue by measuring the change in the resistance of CNT/PDMS while heating it on the hot plate. Figure S10 shows the resistance as the temperature changes. We set the temperature range (24–42 °C) of thermal cycles for the CNT/PDMS samples with a width of 5 mm, a length of 40 mm, and a thickness of 1 mm. The temperature range was set by our heating and measurement system. As the cycle proceeds, the resistance of the sample decreased, and the change in the resistance eventually stops. The decreased resistance is due to the closer interconnections between the CNTs by shrinkage of PDMS during heating.

#### Biocompatibility

We examined the cytotoxicity of the CNT/PDMS composite with human primary keratinocyte HaCaT cells. The viabilities of cells cultured with 4-wt% CNT in the PDMS showed 84.36 ± 38.90% live cells, compared to those on the culture plate (Figure S4). However, PDMS has a highly hydrophobic surface, and the cells tended to attach less strongly to the PDMS surface than to the polystyrene plate. After recalculating the viability using the optical density of PDMS as the control value, the rates of cell viability were 83.38 ± 29.68, 84.36 ± 38.90, and 57.24 ± 26.94% for the 1, 6, and 8 wt% CNT/PDMS composites, respectively. The cytotoxicity of the solution eluted from the 4 wt% CNT/PDMS nanocomposites was compared with that from PDMS, and no significant changes were observed in the statistical values. Thus, CNT/PDMS with CNT contents of less than 6 wt% has biocompatibility equivalent to that of PDMS, as no cytotoxicity was reported^45^ (Figure S4).

Various *in vivo* experiments have been performed to investigate the effects of CNTs in dermal contact. Murray *et al*. found that the exposure of mouse skin to impurities on CNTs (trace metals) caused oxidative stress, depletion of glutathione, increased dermal cell numbers, and skin thickening. Thus, CNT purification is important for reducing toxicity of the dermally administered materials^46^. Toh *et al*. reported that ultrasonication significantly reduced toxicity by promoting the release of metallic impurities into the solution^47^. Thus, the ultrasonication and IPA in our process should minimize such issues. Moreover, in our system, whole bundles or segments of CNTs are tightly strained in the polymer matrix. The tubes protruding from the composite, of <1 μm in length, can sense bioelectric currents on the skin surface, but cannot penetrate the epidermis.

### Applications of the CNT/PDMS composite

#### Flexible electronic circuit

**Figure 5:**
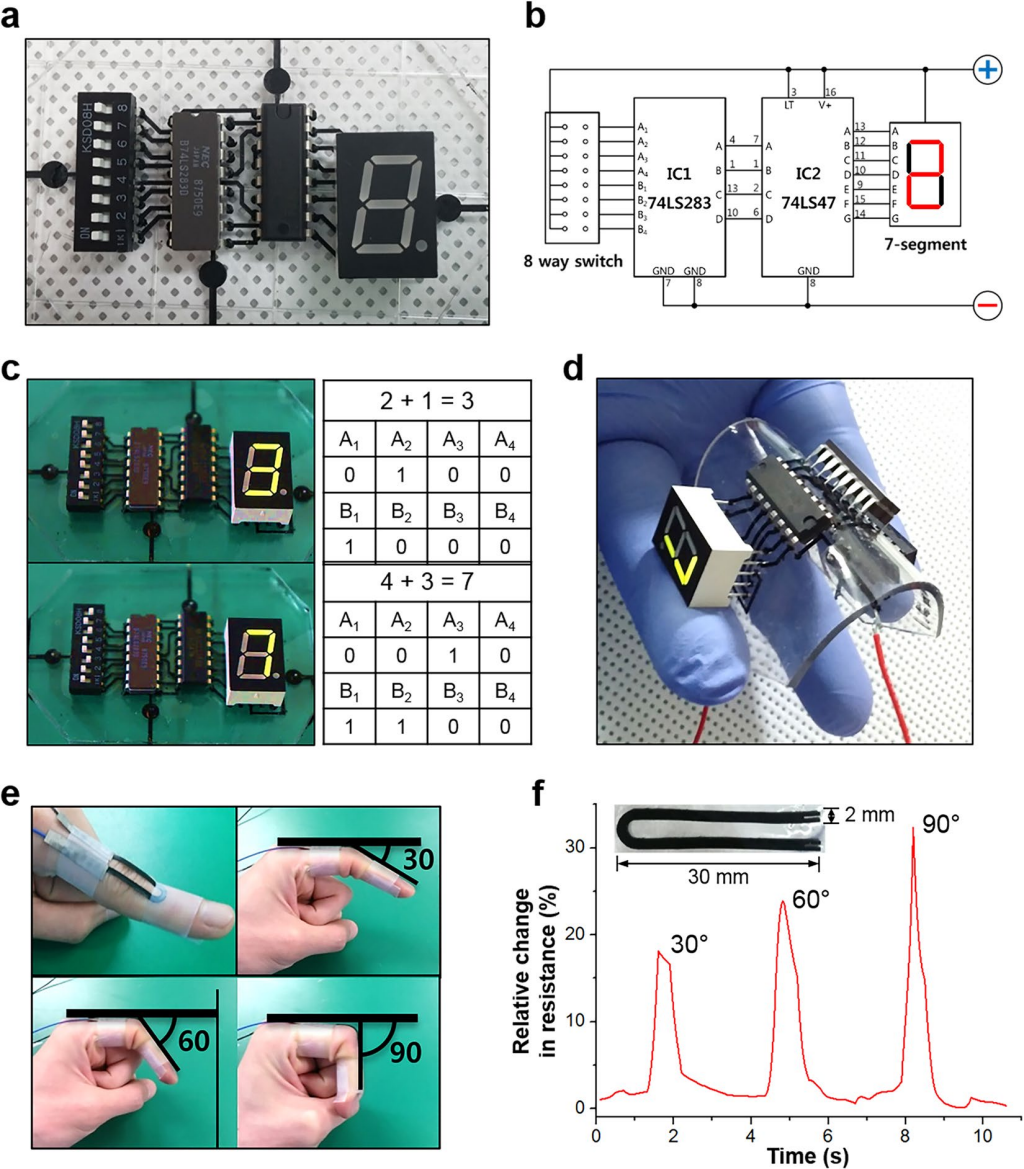
Applications using CNT/PDMS composite. (**a**) Assembled flexible electronic circuit, (**b**) schematic of electronic circuit, (**c**) working circuit and truth table, (**d**) circuit working during bending, (**e**) photograph of finger attached to CNT/PDMS sensor, and (**f**) graph of relative change in resistance over time during finger bending.

We made metal-free flexible circuits comprising PDMS substrates and the CNT/PDMS nanocomposite, as shown in Fig. 5a. Electrical coupling (mounting) between a metal and a polymer has many difficulties, and many studies are ongoing to solve this problem. Our study solved this problem simply by inserting the electric components into the CNT/PDMS line when producing the flexible electronic circuit, as shown in Figure S8e. The connection between the electronic components and the CNT/PDMS composite had no problem with the operation of a flexible electronic circuit bent on top of the finger. The electric components are well-connected, as demonstrated (Fig. 5c) by changing the digits (Sum: A + B) of the seven-segment LED display with different inputs (A and B in Figure S9). Even when the circuit is bent by more than 90°, the circuit works without decreasing the brightness of the LEDs (Fig. 5d). The substrate (PDMS + CNT/PDMS line, thickness: 1 mm) without electronic components can be bent to 2 mm in diameter.

#### Strain sensor

The CNT/PDMS composite was then tested as a strain sensor. The U-shaped strain sensor (width of 2 mm and thickness of 0.5 mm) with wires connected on one side was attached to the joint of a forefinger, as shown in Fig. 5e. The change in the resistance is recorded with a multimeter when the finger is bent to angles of 30, 60, and 90°. When the nanocomposite is stretched under a tensile strain, the interconnections and spacing among the CNTs are changed. The CNTs were separated, leading to a loss of contact points and widening of the intertubular distances^48^. Some CNTs were broken owing to tensile failure as a result of mechanical deformation (the red circles in Figure S5c)^49^. These broken CNTs disrupt the ability of electron transfer in the conductive network and cause the overall resistance to change. We confirmed a linear trend in in the change in the resistance with the degree of bending of the finger (Figure 5f). Moreover, the sensitivity was calculated as the change in the resistance divided by the change in the total sensor length and is 0.59–0.64 Ω/mm. After the bending test, the base resistance was recovered with only 3% relative change, and the duration of the base resistance was influenced by human body vibrations.

#### Electrode for biopotential acquisition

**Figure 6:**
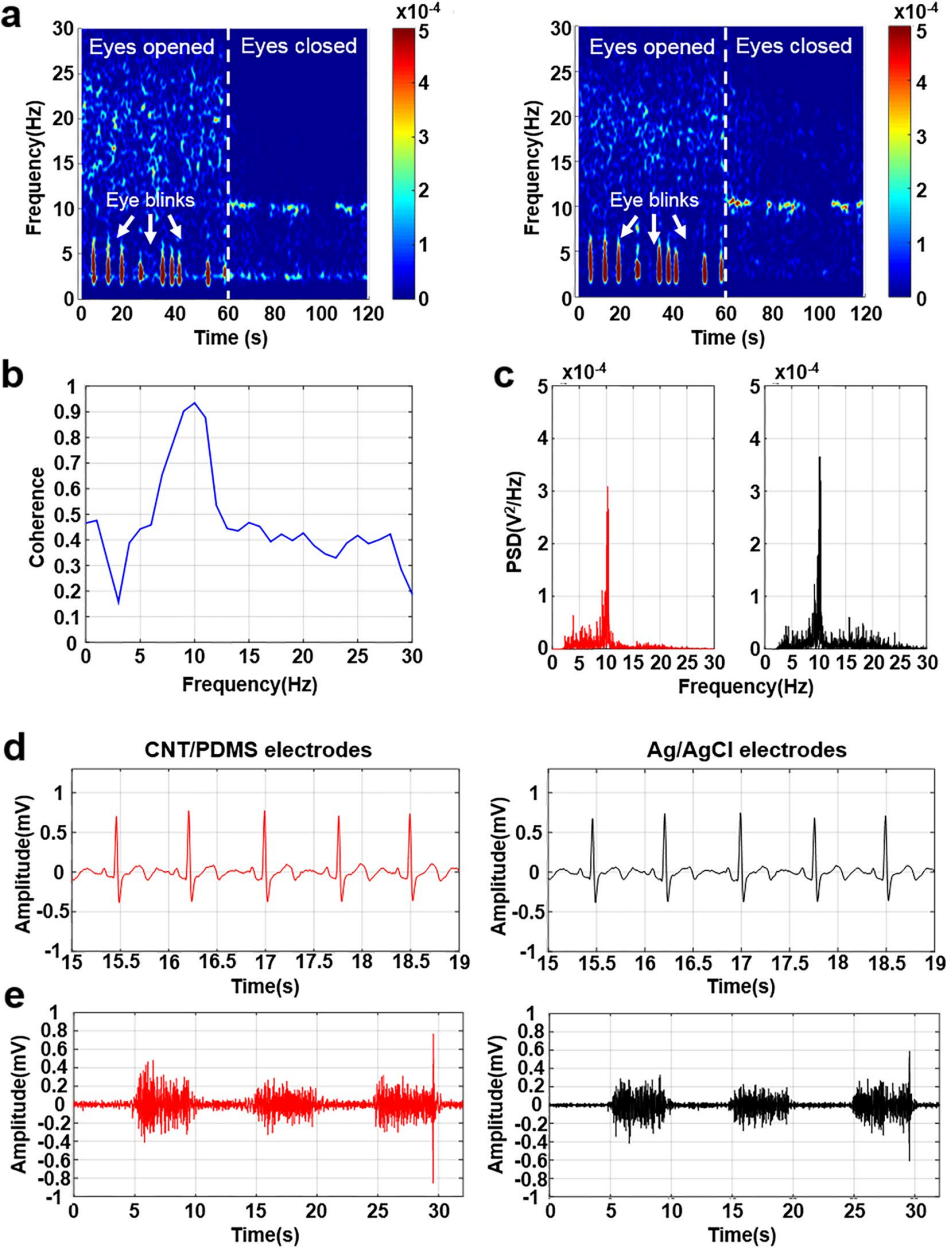
Bio-potentials from CNT/PDMS sensor and commercial sensor. (EEG: a–c, ECG: d, EMG: e). (**a**) Spectrogram analysis of alpha rhythm, (**b**) coherence result of CNT/PDMS electrode and conventional Ag/AgCl electrode during closed-eye period in alpha wave detection test, (**c**) power spectral density of alpha rhythm detection, (**d**) results of ECG measurement, and (**e**) results of EMG measurement. (Red line: CNT/PDMS electrode; black line: Ag/AgCl electrode.)

Electroencephalogram (EEG) measurements were performed at locations Fp2 (right forehead) and A2 (right earlobe) in the 10–20 system^50^. Alpha-rhythm waves are specified as changes in the frequency domain of the alpha band of 8–12 Hz when the subject is relaxed. As the subjects opened and closed their eyes for 60 s, EEGs were recorded with no noise from body movement artifacts or external sources. Artifacts from the eye movement were measured with open eyes. After the subject closed their eyes, dominant signal changes in the alpha band were measured (Fig. 6a). The coherent closed-eye case was calculated using both CNT/PDMS electrodes and conventional electrodes for statistical analysis. The coherence near 10 Hz is over 0.93 when both electrodes record the alpha rhythm simultaneously (Fig. 6b). Figure 6c shows that the power of the conventional Ag/AgCl electrode was larger than that of the developed CNT/PDMS electrode. However, the CNT/PDMS electrode shows good performance in the EEG experiment.

A 60-s electrocardiogram (ECG) measurement was recorded from three electrodes attached to the left and right arm of the subject (Fig. 6d). Electromyogram (EMG) measurements were conducted on the right arm surface for 30 s^51^. The subject repeated an applied strain to the right arm three times for 5 s each (Fig. 6e). The average correlation coefficients from the ECG and EMG signals are 0.97 and 0.85, respectively, suggesting little difference in biopotential measurements performed using CNT/PDMS or Ag/AgCl electrodes. The CNT/PDMS-based electrode could effectively measure changes in the biopotential. To verify the biocompatibility of the CNT/PDMS material with the skin, the subjects wore the CNT/PDMS electrodes continuously for 7 subsequent days. No irritation, erythema, or inflammation effects were reported. Therefore, the CNT/PDMS electrode could be effectively used for long-term continuous biopotential recording, as the material shows good biocompatibility with human skin.

## Conclusion

We have developed an efficient and effective system for creating highly homogeneous dispersions of CNT nanocomposite materials in PDMS elastomers: our proposed method is simple (5-step process), fast (~6–8 h), and cost-effective (~$1–5/g). Curing the elastomer creates a highly conductive and stretchable material with sensitive properties for wearable electronic applications. The SEM and Turbiscan results demonstrated that the CNTs were homogeneously distributed in the PDMS polymer matrix. The homogeneously dispersed nanotubes in the matrix could enhance the mechanical and electrical properties of the tested CNT/PDMS nanohybrids.

The CNT/PDMS nanohybrids advantages included high conductivity (with sheet resistances <20 Ω/sq), excellent tensile stress of ~3.65 MPa, high flexibility (more than 90°) and elasticity (more than 45% yield strain), and good strain sensitivity and stability (gauge factor reaching 10,000 cycles). This is demonstrated to create functional materials that are used for effective bio-signal monitoring of brain, heart, and muscle signals via EEG, ECG, and EMG, as well as devices with incorporated integrated flexible circuits and strain sensors. This low-cost rapid fabrication method for CNT/PDMS nanohybrids provided promising materials for use in wearable electrical stimulation and detection devices, and other applications in biomedical applications, particularly considering the growing global interest in ubiquitous healthcare.

## Methods

### Materials

Multi-walled carbon nanotubes (MWCNTs, CM-95, >95.0%, 10–20-nm outer diameter, 10–20-μm length) were obtained from Hanwha Nanotech (Seoul, Republic of Korea). All PDMS components were purchased from Dow Corning (Midland, MI, USA). All chemicals, including IPA and other organic solvents, were obtained at high-performance liquid chromatography (HPLC) grades with >99.9% purities from Sigma-Aldrich (St. Louis, MO, USA).

### Preparation of the CNT/PDMS Nanocomposites

Pristine MWCNTs were first dispersed in IPA with a 100:1 weight ratio and ultrasonicated for 30 min to obtain single CNTs dispersed in excess IPA solution. Then, 20-wt% low-viscosity (100 cSt) silicone fluid (MEP) was added to the dispersion and ultrasonicated for 10 min. To obtain a homogeneous dispersion, 80 wt% of PDMS-A was added and ultrasonicated for 10 min. After IPA was evaporated from the dispersion using a hot plate at 55 °C, the crosslinker PDMS-B was added and vigorously mixed. A vacuum desiccator was used to remove the bubbles remaining from the mixing process. The blend was cast in a mold and cured in an oven for 2 h at 80 °C.

### Characterization of CNT/PDMS Nanocomposites

We measured the stability of the aqueous CNTs dispersions using a stability analyzer (Turbiscan™ Lab, Formulaction Inc., Toulouse, France) at 25 °C for 24 h. Each dispersion was poured into a 30-mL cylindrical glass cell to a height of 55 mm, and the stability was monitored by measuring the transmission (T) and backscattering (BS) of a pulsed near-infrared (NIR) light at 880 nm. The transmittance detector received the light that passed through the dispersion at an angle of 180° relative to the source, while the backscattering detector received the light scattered backward by the dispersion at an angle of 45°. The detection head scanned the entire height of the sample, acquiring the transmittance and backscattering data in steps of 40 μm for 24 h. Infrared spectra were recorded from the solid specimens in the frequency range of 400 to 4000 cm^−1^.

### Chemicophysical Properties of CNT/PDMS Films

Comparative analysis of different substrates was performed by contact angle analysis using a Phoenix 300 instrument (Surface Electro Optics, Suwon, Republic of Korea) for wettability analysis, Raman spectrometry at ~532 nm using a LabRam Aramis IR2 (Horiba, Kyoto, Japan), ATR-FTIR using a Varian 640-IR instrument (Varian, Palo Alto, CA, USA), XRD using an Xpert MRD System (Philips, Amsterdam, the Netherlands) for chemical quantification, and field-emission scanning electron microscopy (FE-SEM) using a MIRA II LMH microscope (TESCAN, Brno, Czech Republic) for detailed morphologic analysis. For SEM analysis, the samples were sputter-coated with 10 nm of gold before analysis. The tensile stress and strain of the composite films were measured by the Instron 5966 universal testing machine (Instron, Norwood, MA, USA). The dimensions of the dog-bone-shaped specimens were 100 mm in overall length, 33 mm in narrow section length, 6 mm in gauge length, 3 mm in gauge width, and 1 mm in thickness, according to Die D in the ASTM D412 standard measurement for rubbers and elastomers.

### Analysis of Electrical Properties of CNT/PDMS Composites

To measure the sheet resistance of each composition of CNT/PDMS, we cut circular specimens (20-mm diameter and 1-mm thickness) and used four-point probe sheet resistance/resistivity measurements (CRESBOX, Napson Corp., Tokyo, Japan).

### Measurement of biopotential signals

The study protocol was approved by the Institutional Review Board (IRB, Approval No.1040548-KU-IRB-16-48-A-2) of Korea University, Seoul, South Korea, and all subjects(10 adults, age 20 ~ 30 s, 7 males, 3 female) provided written informed consent. The experiments were carried out in accordance with the approved guidelines of IRB.

## Electronic supplementary material

Supplementary Data
