# Supplementary material for: Simple and cost-effective method of highly conductive and elastic carbon nanotube/polydimethylsiloxane composite for wearable electronics
Source: Sci Rep. 2018 Jan 22;8:1375. doi: 10.1038/s41598-017-18209-w (PMC5778073; doi:10.1038/s41598-017-18209-w)
Supplement: Supplementary file 1 — Supplementary Data [file 41598_2017_18209_MOESM1_ESM.doc]

**Simple and cost-effective method of highly conductive and elastic carbon nanotube/polydimethylsiloxane composite for wearable electronics**

Jeong Hun Kim^1^, Ji-Young Hwang^2,*^, Ha Ryeon Hwang^1^, Han Seop Kim^1^, Joong Hoon Lee^1^, Jae-Won Seo^3^, Ueon Sang Shin^3^, and Sang-Hoon Lee^1,2,*^

^1^KU-KIST Graduate School of Converging Science and Technology, Korea University, Seoul, 02841, Republic of Korea

^2^Department of Biomedical Engineering, College of Health Science, Korea University, Seoul, 02841, Republic of Korea

^3^Department of Nanobiomedical Science and BK21 Plus NBM Global Research Center for Regenerative Medicine, Dankook University, Cheonan 31116, Republic of Korea

[^*^jyhwang@korea.ac.kr](mailto:*jyhwang@korea.ac.kr), dbiomed@korea.ac.kr

**Supporting Information**

**Table S1. Summary of CNT/PDMS composite materials by gentle mixture versus sonication.**

| **Samples** | **TurbiScan^a^** | | **TGA (ºC)** | |
| --- | --- | --- | --- | --- |
|  | **Mixture** | **Sonication** | **Mixture** | **Sonication** |
| **CNT/IPA** | US | S | 700 | 620 |
| **CNT/IPA/MEP** | US | S | 410/720 | 420/685 |
| **CNT/IPA/MEP/PDMS-A** | US | S | 400/720 | 430/730 |
| **CNT/IPA/MEP/PDMS-A & -B** | US | S | 400/710 | 420/730 |
| **PDMS-A & -B in IPA** | ND**^b^** | ND | 300/500 | 300/500 |

a: Stable (S) and unstable (US) solution

b: Not determined (ND)

**Table S2.** Data summary of the characteristics CNT/PDMS films according to each CNT percentile.

| **Samples** | **Young’s modulus (MPa)** | **Elongation (%)** | **Sheet resistance (Ω/sq)** | **Electrical conductivity (S/cm)** | **Contact angle (º)** |
| --- | --- | --- | --- | --- | --- |
| **PDMS** | 1.71 ± 0.17 | 118.90 ± 14.66 | ND | ND | 102.20 ± 1.32 |
| **MEP/PDMS**  **(0 wt % CNT/PDMS)** | 1.80 ± 0.05 | 98.95 ± 15.80 | ND | ND | 98.60 ± 3.29 |
| **1 wt % CNT/PDMS** | 2.47 ± 0.06 | 81.84 ± 21.36 | 5225 ± 1755 | 0.003 ± 0.001 | 103.10 ± 1.51 |
| **2 wt % CNT/PDMS** | 2.55 ± 0.12 | 84.41 ± 20.56 | 212.30 ± 30.92 | 0.057 ± 0.009 | 103.00 ± 2.89 |
| **4 wt % CNT/PDMS** | 2.90 ± 0.08 | 70.84 ± 17.07 | 23.670 ± 1.069 | 0.208 ± 0.023 | 108.20 ± 4.13 |
| **6 wt % CNT/PDMS** | 3.07 ± 0.06 | 58.99 ± 9.40 | 13.400 ± 1.253 | 0.377 ± 0.006 | 104.30 ± 2.40 |
| **8 wt % CNT/PDMS** | 3.21 ± 0.16 | 64.77 ± 22.80 | 8.720 ± 1.480 | 0.671 ± 0.021 | 107.90 ± 1.52 |
| **12 wt % CNT/PDMS** | 3.65 ± 0.12^a^ | 44.86 ± 5.71^a^ | 3.433 ± 0.619 | 1.228 ± 0.021 | 117.80 ± 3.15 |
| **20 wt % CNT/PDMS** | ND | ND | 2.026 ± 0.163 | 2.281 ± 0.137 | 128.30 ± 3.81 |

^a^ Data on 10 wt % CNT/PDMS

ND: Not determined

**
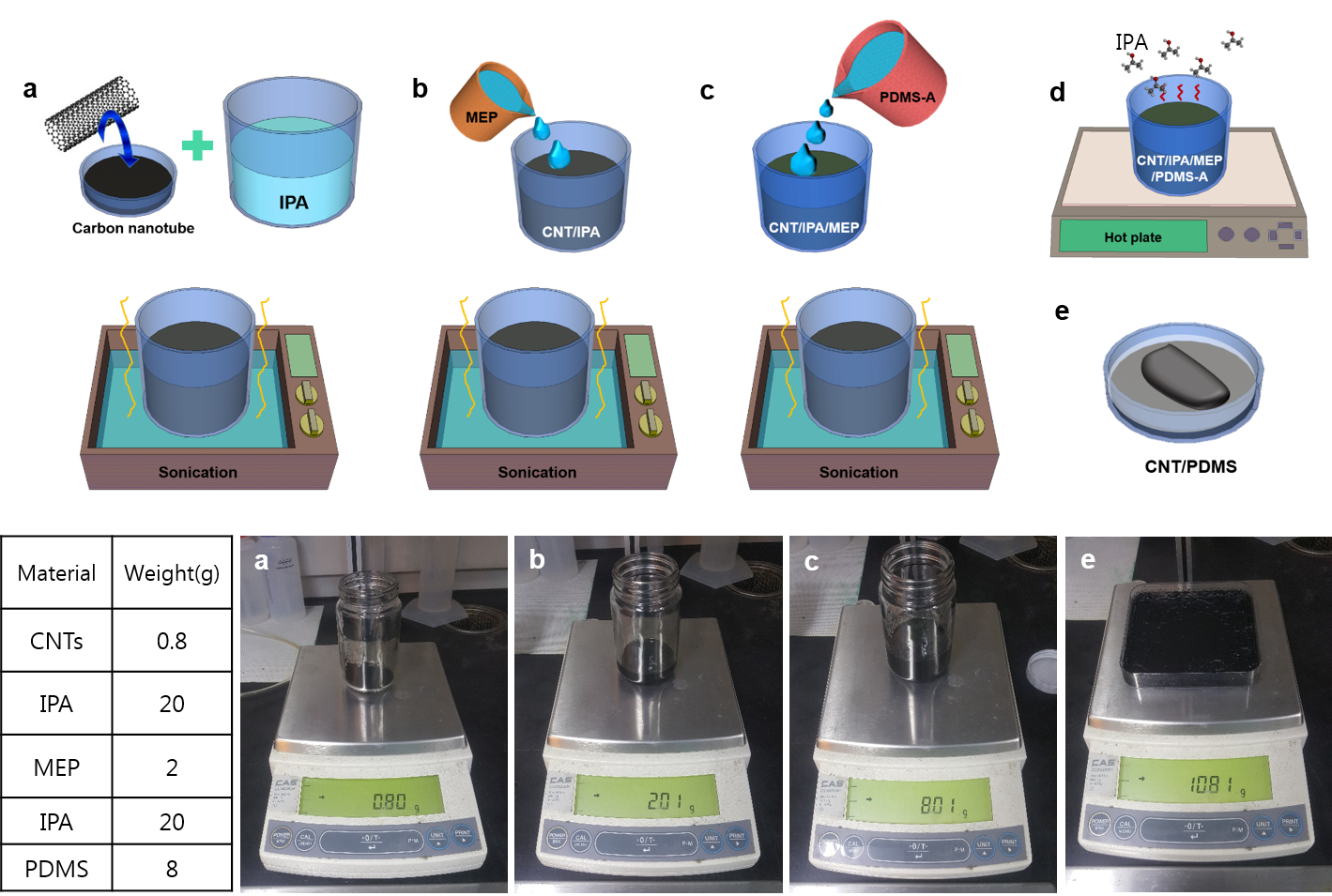
**

**Figure S1**. Process of preparing CNT/PDMS. (a) Pristine MWCNTs were first dispersed in IPA with a 100:1 weight ratio and ultrasonicated for 30 min to obtain single CNTs dispersed in excess IPA solution. (b) A 20-wt% low-viscosity (100 cSt) silicone fluid (MEP) was added to the dispersion and ultrasonicated for 10 min. (c) To obtain a homogeneous dispersion, 80-wt% PDMS-A was added and ultrasonicated for 10 min. (d) IPA was evaporated from the dispersion using a hot plate at 55°C. (e) The crosslinker PDMS-B was added and vigorously mixed. When creating 8-wt% CNT/PDMS, the weights of component materials at each step are indicated by the photographs of the electronic scale.

**
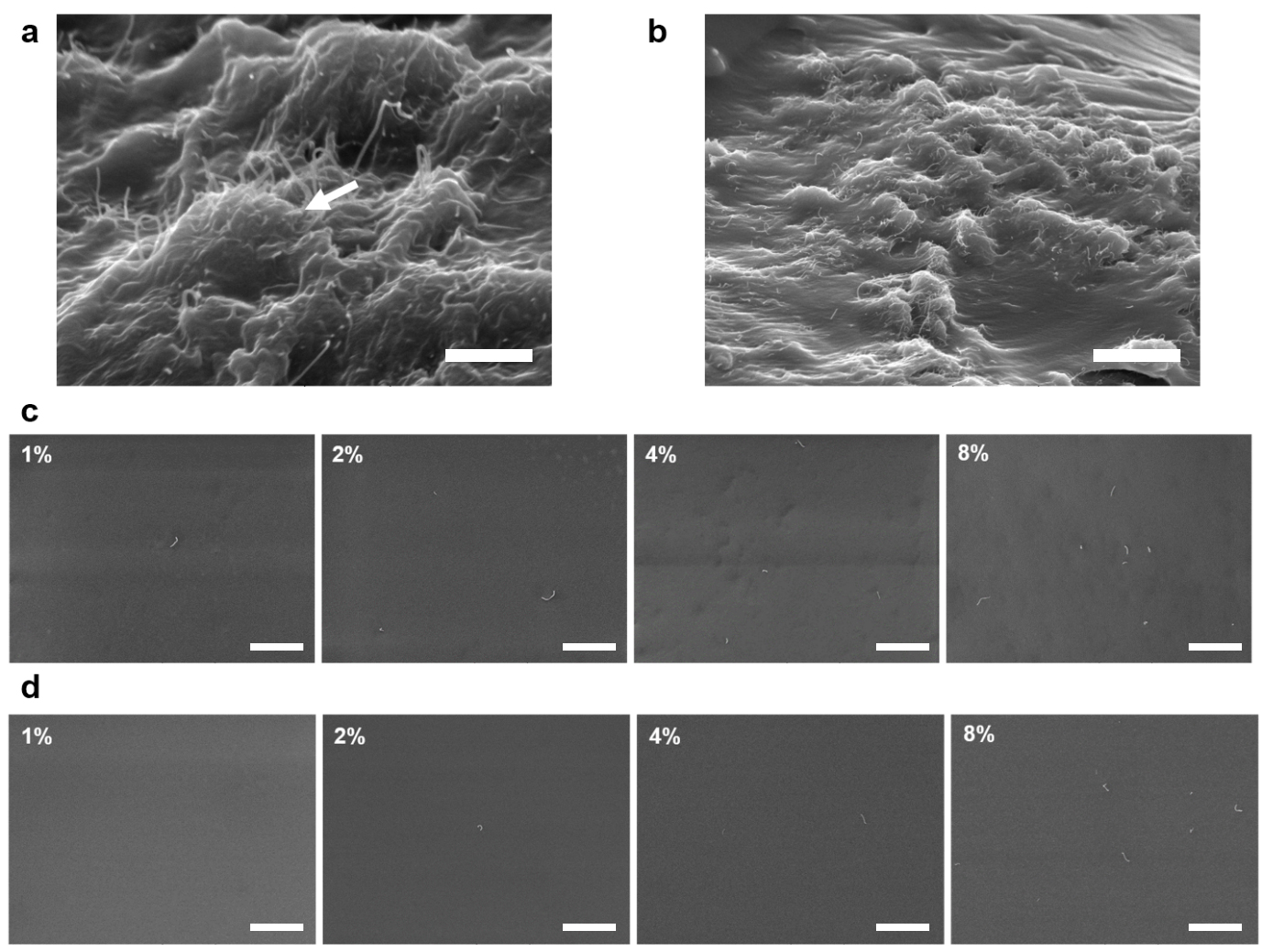
**

**Figure S2**. Images taken by FT-SEM. (a, b) Cross-sectional SEM images of 4-wt% CNT/PDMS films. (scale bar, 1μm) Detachment testing of some loosely bound CNTs of CNT/PDMS pieces by (c) first adhesion and (d) second adhesion of Scotch tape on each percentile of CNT/PDMS films. (scale bar, 5μm)


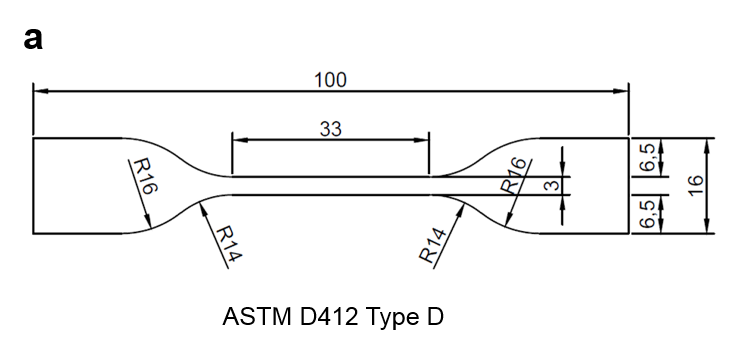

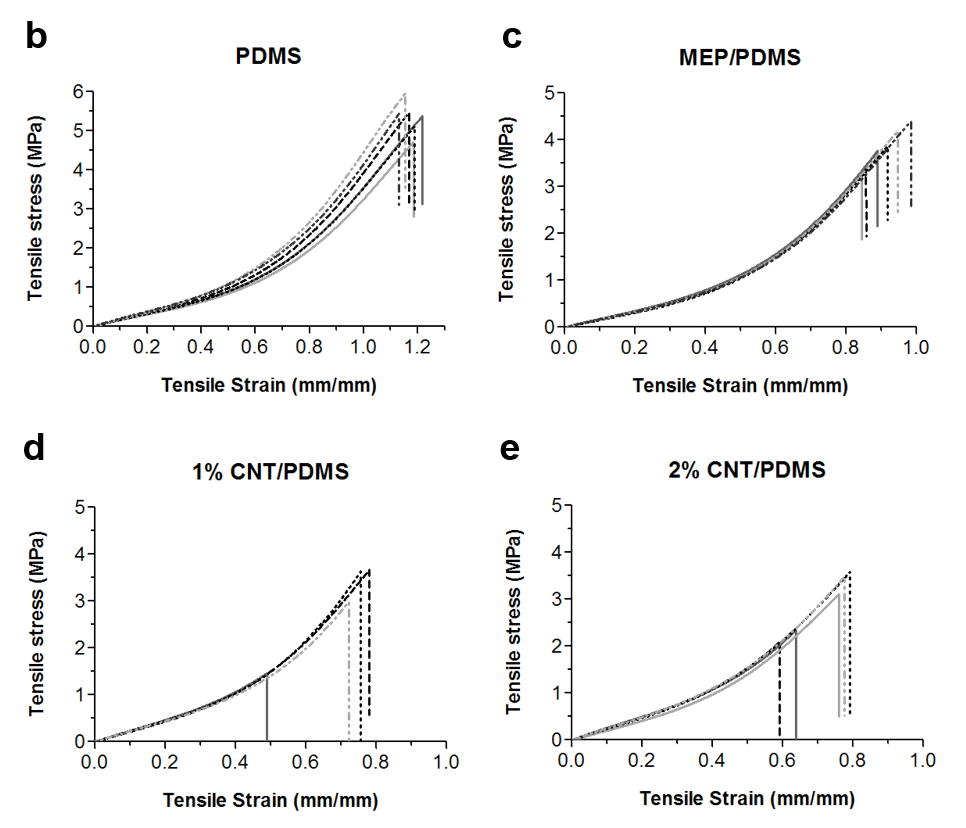

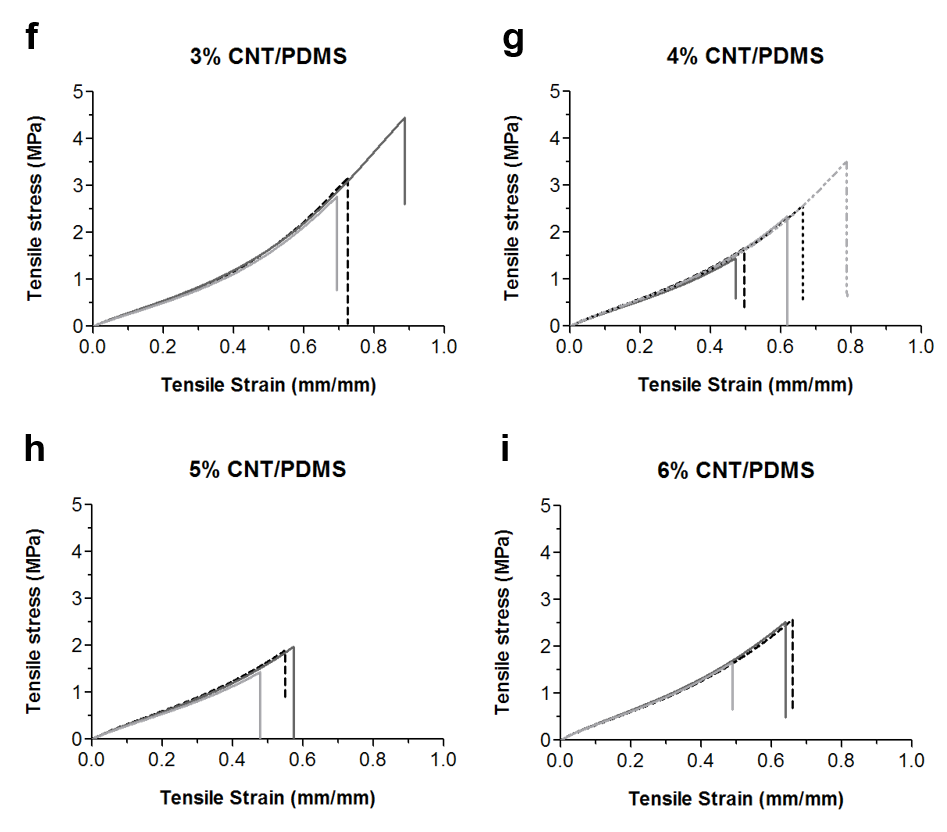


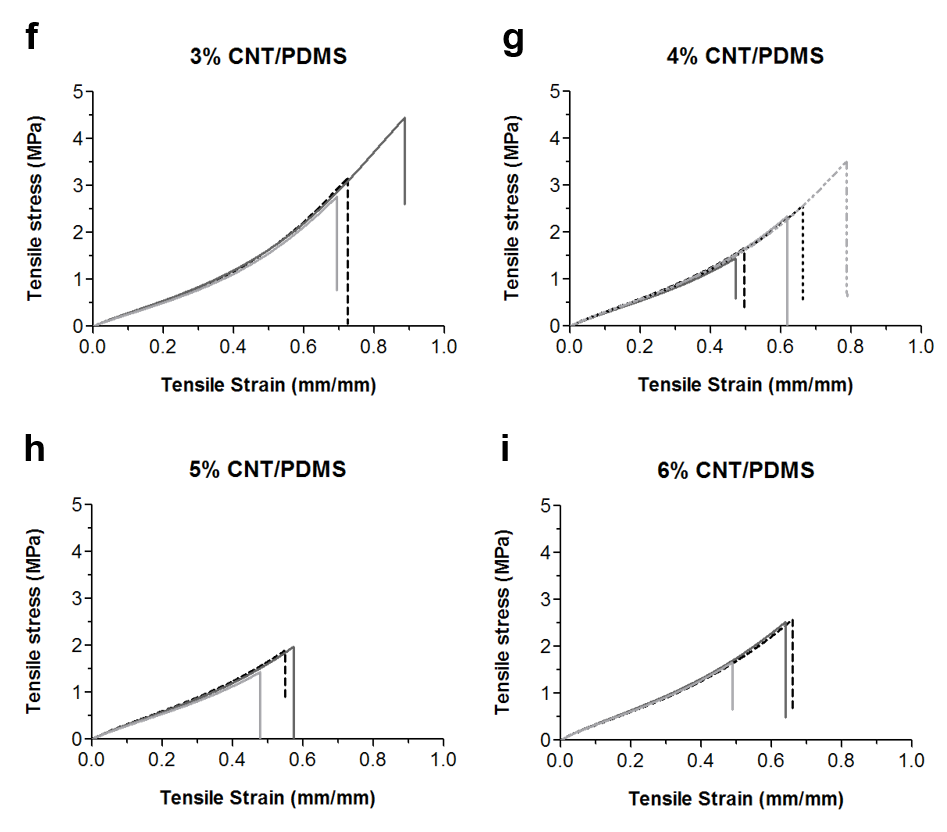


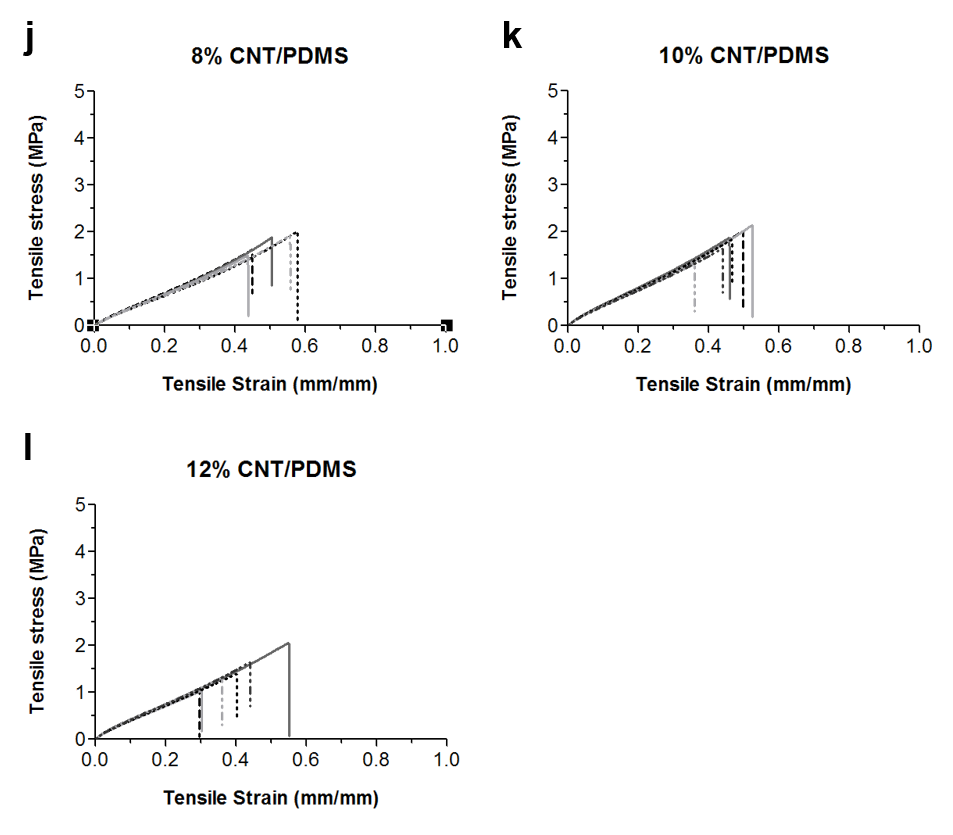


**Figure S3**. Analysis of mechanical properties of each CNT percentile in PDMS. a) ASTM D412 type D, (b–k) tensile stress versus tensile strain of each percentile of CNT in PDMS (n=3–6).

*Cytotoxicity Evaluation*

The *in vitro* cytotoxicity of the CNT/PDMS composites was evaluated using Cell Counting Kit-8 (CCK-8) by following the manufacturer’s instructions (Dojindo Laboratories, Kumamoto, Japan). The human primary keratinocyte cell line, HaCaT, was used after culturing in Dulbecco’s modified Eagle’s medium with high glucose, supplemented with 10% (v/v) fetal bovine serum, 100 U/mL penicillin, and 100 g/mL streptomycin in a humidified incubator with a 5% CO_2_ atmosphere at 37°C.

To investigate cell viability for each percentage of CNT in PDMS, 100-μL HaCaT cell suspensions were seeded with a density of 5 × 10^3^ cells per sample. These were prepared by cutting each CNT/PDMS film into 5-mm-diameter discs, followed by UV irradiation for 30 min for sterilization. The prepared plates were cultured at 37°C for three days and further incubated for 4 h after the addition of 10 μL of CCK-8 solution. The solutions were then transferred into a 96-well plate. The optical density (OD) was determined by measuring the absorbance of 450-nm light using a microplate reader (PerkinElmer, Waltham, MA, USA). The rates of cell viability were calculated by the following equation: Cell viability (%) = (OD_sample_/OD_control_) × 100%, where OD_control_ was the obtained data from a commercial culture plate.

GraphPad Prism 5 (GraphPad Software, USA) was used to analyze, graph, and present the data. Cell viability and quantitative morphology data were evaluated using the analysis of variance (ANOVA) method to test for differences among the groups subjected to different substrate modifying strategies, followed by a comparison of means using Tukey post-hoc analysis or Bonferroni post-tests. Statistical significance was considered at p < 0.05. Each experiment was performed at least three times.


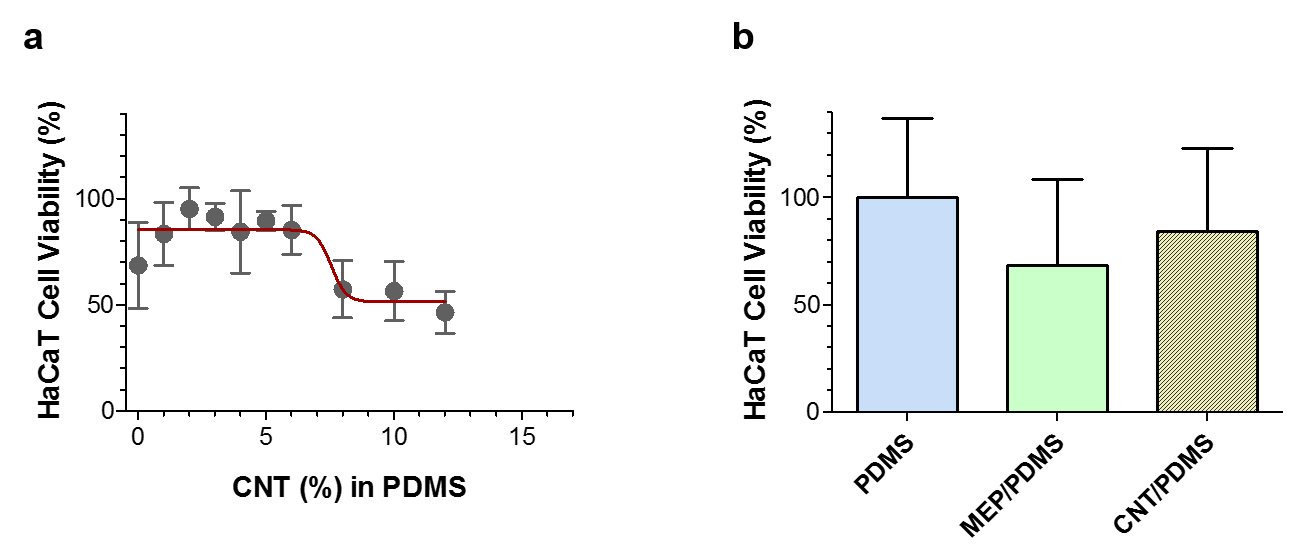


**Figure S4**. Biocompatibility analysis. Cell viability assay of direct culture of a, b) human primary keratinocyte (HaCaT). Error bars represent standard deviations (n=4–8). The red bold lines indicate the curve fitting.


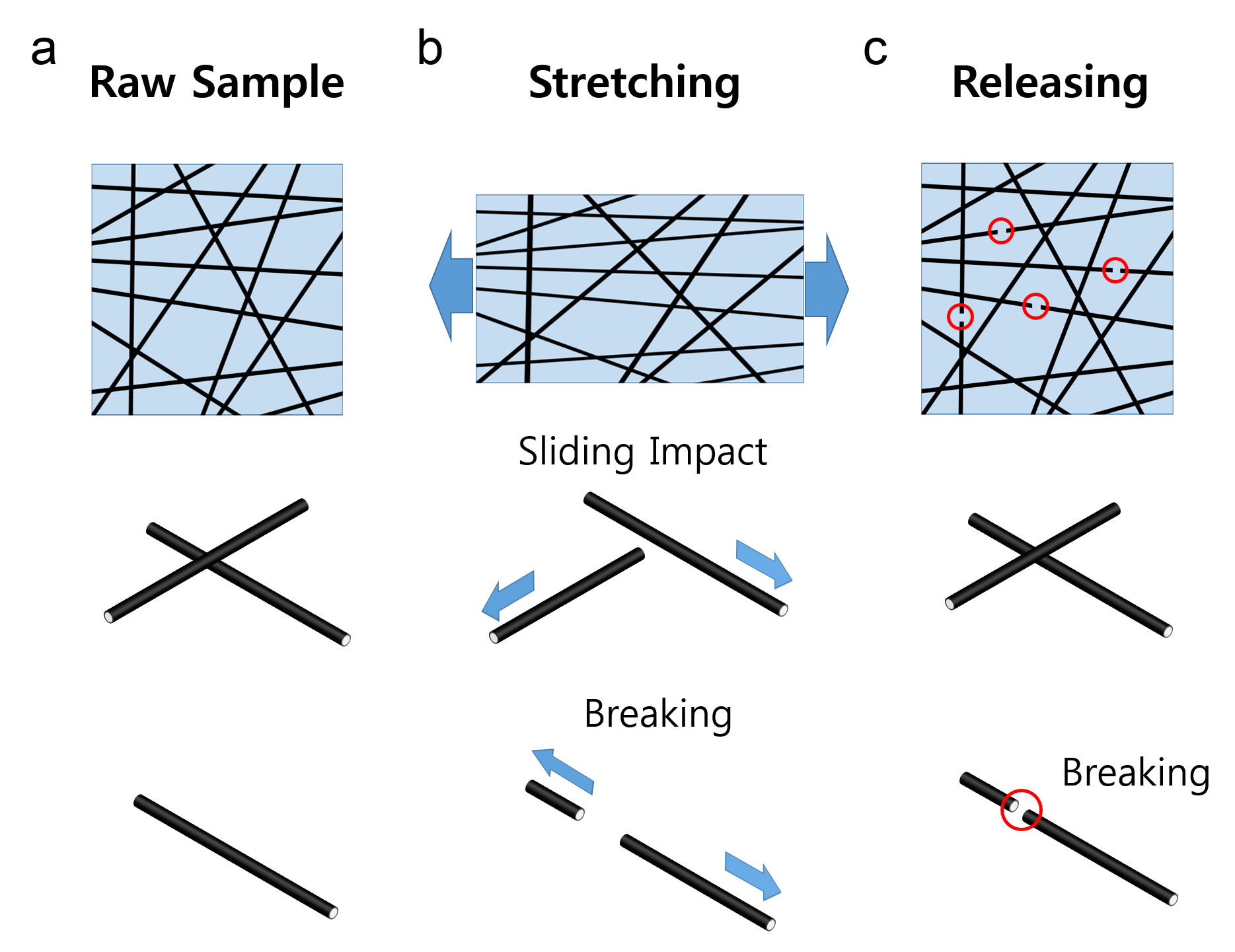


**Figure S5**. Changes in the interconnections and spacing between CNTs. (a) CNTs interact in the composite without a tensile strain. (b) The interconnections and spacings between CNTs change when the nanocomposite is stretched under a tensile strain. (c) CNTs interact in the composite after a tensile strain. Some CNT interactions recover the contact junctions (sliding impact), but several CNTs are destroyed because the tensile force is sufficiently large to cause breakage, as indicated by the red circles.

*Cyclic Strain Testing*

Tensile strains were loaded and unloaded cyclically using a laboratory-made and computer-controlled stretching stage equipped with a stepper motor. The linear X-axis stage (XMSG650-LC24, Misumi, Tokyo, Japan; controller: D212, Suruga Seiki, Shizuoka, Japan) comprised an actuating unit (moving part) and a stationary part, with an attached lab-produced vise to fix a specimen measuring 5 × 50 × 1 mm. Before the stretching operation, the sample was clamped to the stage with a stretching portion measuring ~30 mm in length. The sample was connected to copper tape outside the stretching part. A digital multimeter (34401A, Agilent, Santa Clara, CA, USA) was connected to the copper tape and recorded the resistance of the CNT/PDMS specimen throughout the strain test.

*Strain Sensor*

We fabricated a strain sensor with 8-wt.% CNT/PDMS and a mold. The mold was carved with an intaglio U-shaped pattern. Uncured CNT/PDMS was pushed into the intaglio pattern, baked at 80°C for 1 h, and separated in cured form. The strain sensor was designed in this shape to suit the human finger joint. The sensor (30-mm length, 2-mm width, 0.5-mm thickness) was easily connected to wire at the end of the U-shape. Using the digital multimeter and a computer, we recorded the resistance change of the strain sensor on the human forefinger with bending to 30, 60, and 90°.


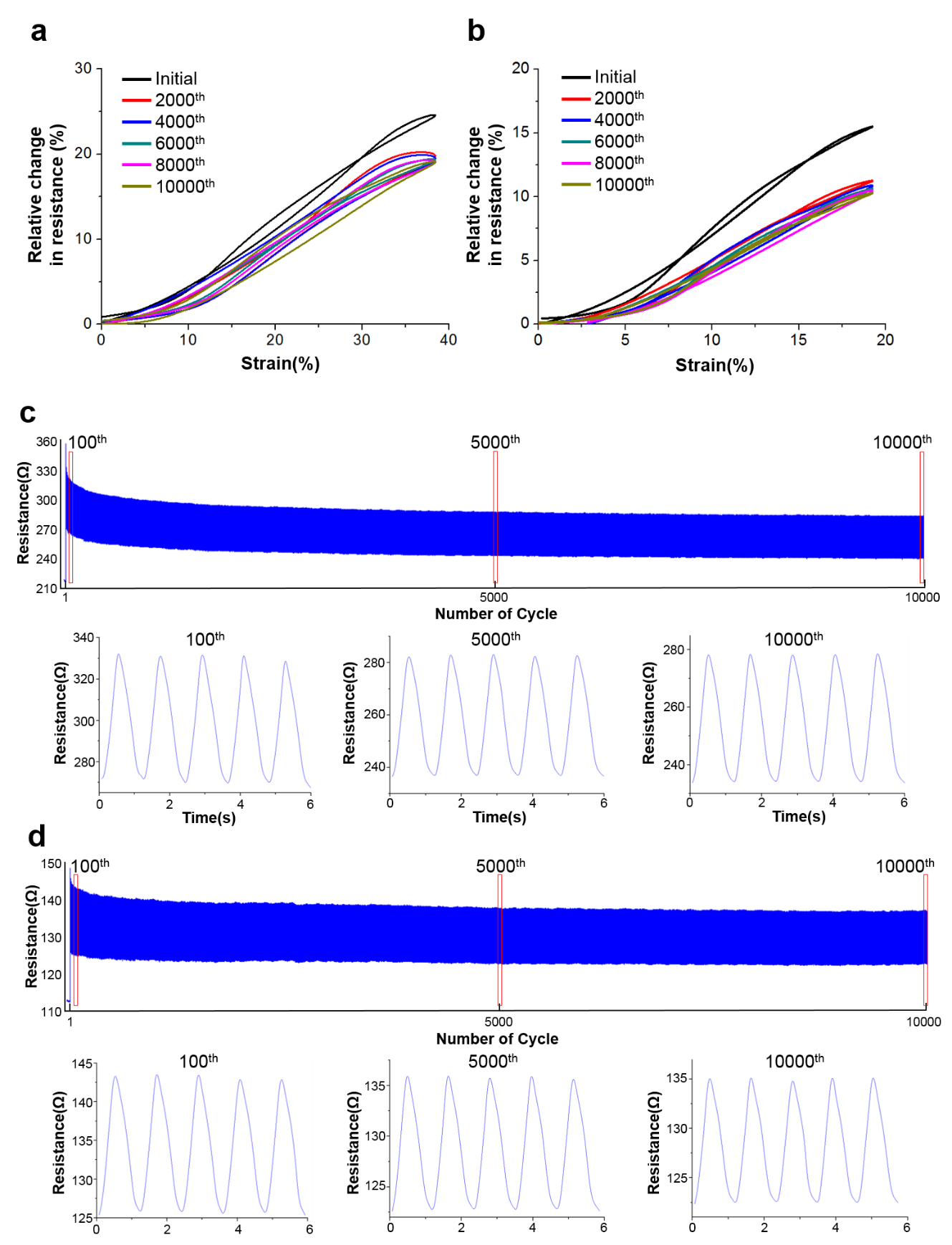


**Figure S6**. Strain cycle test with difference weight percent CNT/PDMS. Graph of measuring relative change in resistance during 10,000 times cycle test with a) 8-wt% and b) 12-wt% CNT/PDMS. Cycle test with 10000 cycles using c) 8-wt% and d) 12-wt% CNT/PDMS. The graphs show five cycles around the 100^th^, 5000^th^ and 10000^th^ cycles.

*Fabrication of CNT/PDMS Electronic Circuits*

We fabricated a master template for a four-bit adder electrical circuit with a switch on a silicon wafer using SU-8 (SU-8 100, Microchem Corp., Newton, MA, USA) and photolithography techniques. The master pattern was 500-µm thick and 1-mm wide. PDMS was poured over the master pattern for the replica molding process. The 4-wt.% CNT/PDMS nanocomposite was pushed into the patterned lines of the PDMS circuit. The circuit consisted of dual in-line package (DIP) commercial chips. The four-bit full adder (SN74LS283, Texas Instruments, Dallas, TX, USA) was used, and a seven-segment display and binary-coded decimal (BCD) to a seven-segment decoder (SN74LS48, Texas Instruments) were used to display the sum of two inputs. An eight-way DIP switch was used for two binary inputs. Each four-bit adder in the switch was used as a hexadecimal input. To fix and connect the integrated circuit (IC) chip to the CNT/PDMS line, through-holes were punctured in the PDMS layer, and IC chip pins were inserted into the holes in the CNT/PDMS composite. Finally, the IC chip pins were connected electrically and mechanically using cured CNT/PDMS composite as a conductive paste. We then electrically connected the terminals of the CNT/PDMS lines to a power supply (UP-100D, Unicorn, Republic of Korea).


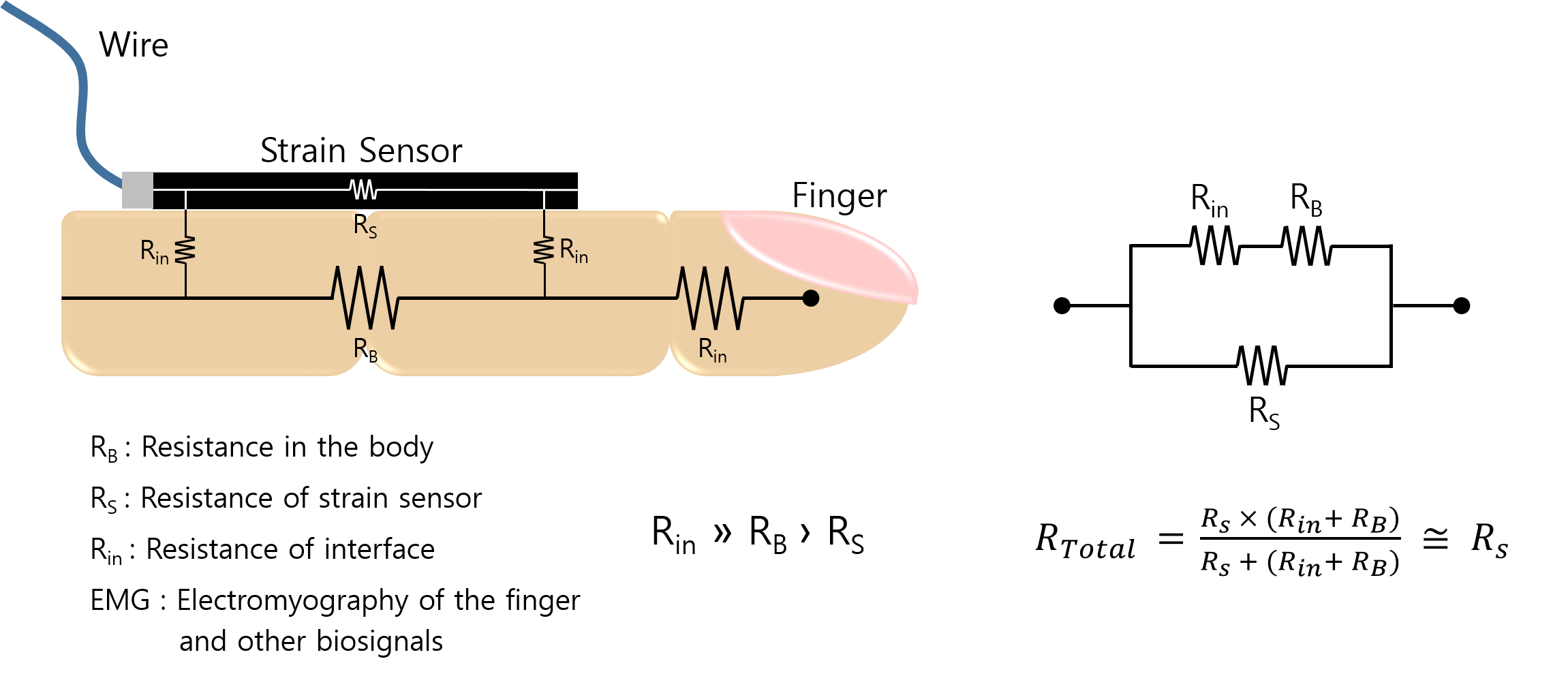


**Figure S7**. Schematic and formula of the electrical relationship between the strain sensor and the finger.


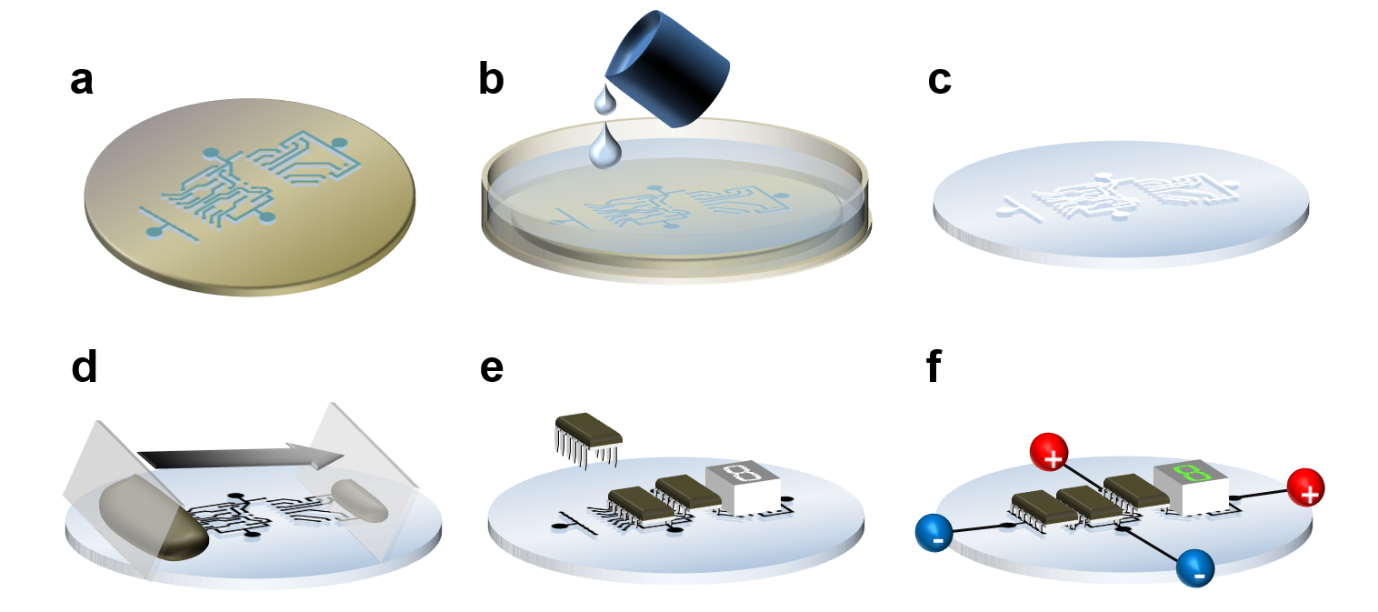


**Figure S8**. Fabrication of flexible electric circuit. a) Fabrication SU-8 master mold using MEMS technology, b) pouring uncured PDMS on the SU-8 master mold, c) curing PDMS on the hotplate at 80°C for 2 h and then separating PDMS from SU-8 master mold, d) push CNT/PDMS composite in the intaglio PDMS channel, e) electronic components are embedded in CNT/PDMS channel, and f) power supply connection.


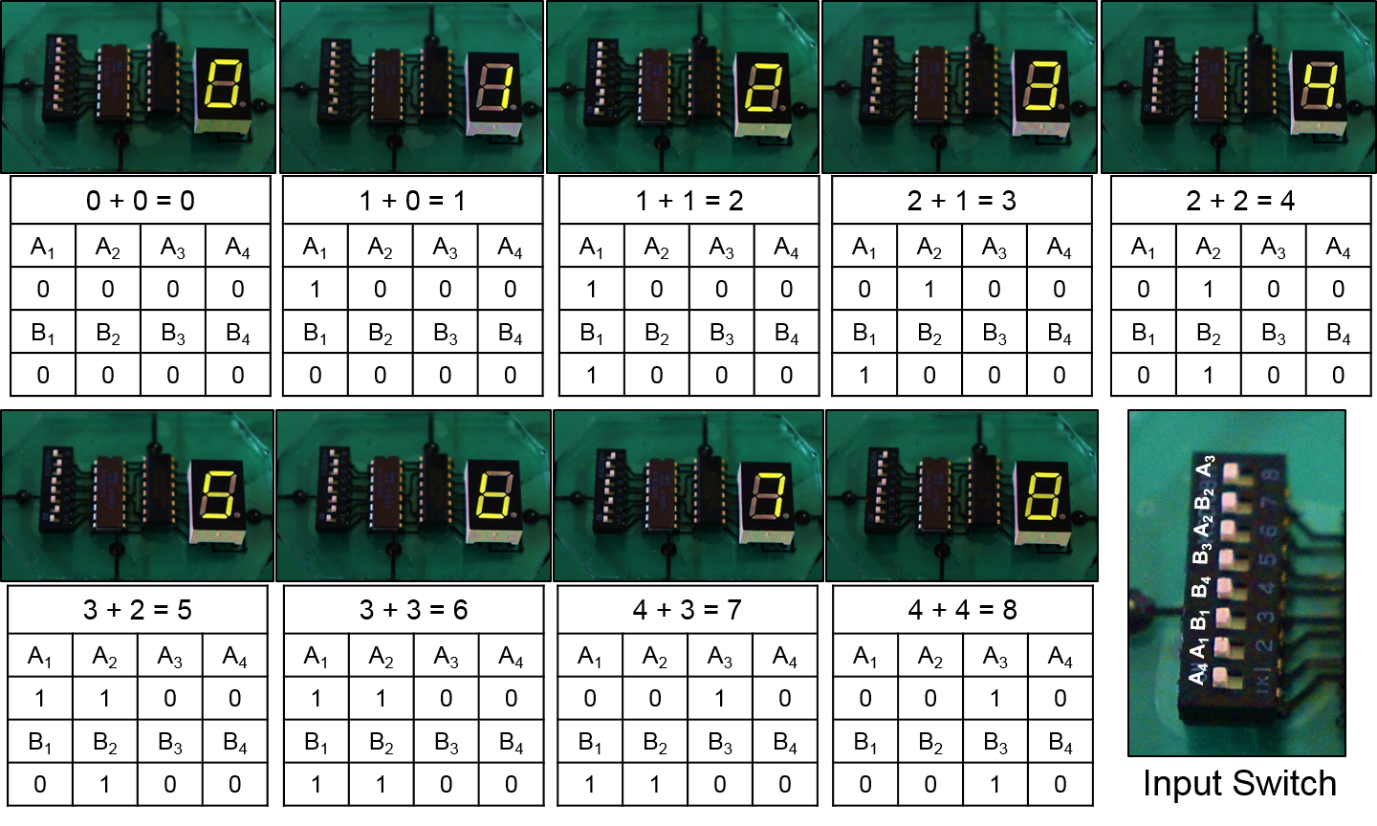


**Figure S9**. Working logic circuit and truth table. 2 Inputs (A, B) in a switch have 4-bit inputs. Here, X_1_ (i.e., A_1_, B_1_) is decimal number 1 (or 2^0^), X_2_ is decimal number 2 (or 2^1^), X_3_ is decimal number 4 (or 2^2^), and X_4_ is decimal number 8 (or 2^3^).


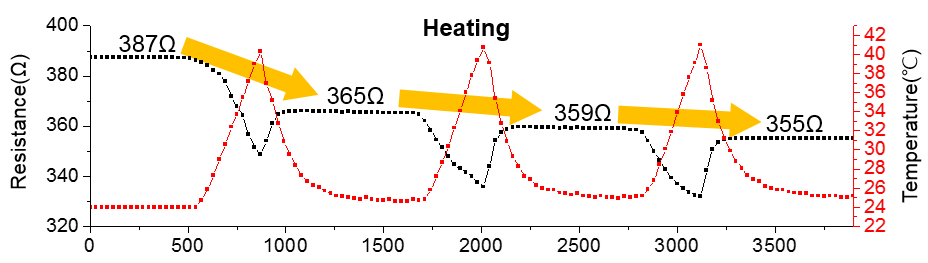


**Figure S10**. Graph of the resistance and temperature changes.
